# Association between periodontal disease and mortality in people with CKD: a meta-analysis of cohort studies

**DOI:** 10.1186/s12882-017-0680-9

**Published:** 2017-08-16

**Authors:** Jian Zhang, Hong Jiang, Min Sun, Jianghua Chen

**Affiliations:** 1 Kidney Disease Center, The First Affiliated Hospital, College of Medicine, Zhejiang University, Hangzhou, 310003 People’s Republic of China; 2 Kidney Disease Immunology Laboratory, The Third Grade Laboratory, State Administration of Traditional Chinese Medicine of PR China, Hangzhou, People’s Republic of China; 3 Key Laboratory of Multiple Organ Transplantation, Ministry of Health, Key Laboratory of Nephropathy, Hangzhou, Zhejiang Province People’s Republic of China; 4 Department of General Surgery, Taihe Hospital, Hubei University of Medicine, Shiyan, 442000 People’s Republic of China

**Keywords:** Cohort studies, meta-analysis, Mortality, Periodontal disease, Outcomes

## Abstract

**Background:**

Periodontal disease occurs relatively prevalently in people with chronic kidney disease (CKD), but it remains indeterminate whether periodontal disease is an independent risk factor for premature death in this population. Interventions to reduce mortality in CKD population consistently yield to unsatisfactory results and new targets are necessitated. So this meta-analysis aimed to evaluate the association between periodontal disease and mortality in the CKD population.

**Methods:**

Pubmed, Embase, Web of Science, Scopus and abstracts from recent relevant meeting were searched by two authors independently. Relative risks (RRs) with 95% confidence intervals (CIs) were calculated for overall and subgroup meta-analyses. Statistical heterogeneity was explored by chi-square test and quantified by the I^2^ statistic.

**Results:**

Eight cohort studies comprising 5477 individuals with CKD were incorporated. The overall pooled data demonstrated that periodontal disease was associated with all-cause death in CKD population (RR, 1.254; 95% CI 1.046–1.503; *P* = 0.005), with a moderate heterogeneity, I^2^ = 52.2%. However, no evident association was observed between periodontal disease and cardiovascular mortality (RR, 1.30, 95% CI, 0.82–2.06; *P* = 0.259). Besides, statistical heterogeneity was substantial (I^2^ = 72.5%; *P* = 0.012). Associations for mortality were similar between subgroups, such as the different stages of CKD, adjustment for confounding factors. Specific to all-cause death, sensitivity and cumulative analyses both suggested that our results were robust. As for cardiovascular mortality, the association with periodontal disease needs to be further strengthened.

**Conclusions:**

We demonstrated that periodontal disease was associated with an increased risk of all-cause death in CKD people. Yet no adequate evidence suggested periodontal disease was also at elevated risk for cardiovascular death.

**Electronic supplementary material:**

The online version of this article (doi:10.1186/s12882-017-0680-9) contains supplementary material, which is available to authorized users.

## Background

In the past decade, chronic kidney disease (CKD) has received mounting attention as a leading public health problem worldwide [1]. It is well established that people with CKD are more susceptible to cardiovascular morbidity and mortality compared to the general population, especially for individuals have advanced CKD requiring renal replacement therapy [2–4]. It estimates that approximately 10–20 of every 100 people treated with dialysis die each year [5]. Cardiovascular diseases are thought to be the main cause of death, accounting for 40% of all-cause mortality for this population [6]. However, traditional risk factors are inadequate to decipher the heightened death risk and the high prevalence of cardiovascular disease. Consequently, nontraditional risk factors have also been widely investigated to explain this phenomenon. Among them, chronic inflammation, defined as persistently high levels of inflammatory biomarkers, is highly prevalent and may contribute to cardiovascular diseases in hemodialysis patients [7].

Periodontal disease is a common group of inflammatory diseases caused by interaction between gram-negative periodontal bacterial species and components of the host immune response [8]. Reportedly, about half of adult people around the world suffer from severe periodontitis, which will finally lead to tooth loss [9]. Besides, previous studies suggested that poor periodontal health is more prevalent and severe in adults with CKD [10]. Although links of periodontal disease with elevated all-cause and cardiovascular mortality were extensively ascertained in the general population [11, 12], the effects of periodontal disease on mortality in CKD people have not been definitively demonstrated.

Up to now, individual study with respect to the relationship between periodontal disease and premature death in people with CKD was not convincing enough, which merely offered imprecise and inconsistent risk estimates [13–15]. In 2009, Kshirsagar et al. performed a retrospective study based on 168 adult hemodialysis patients, and firstly found that moderate-severe periodontal disease was significantly associated with increased risk of cardiovascular death, even after adjustment of age, gender, center and dialysis vintage, smoking status and history of diabetes mellitus or hypertension [14]. While the association with all-cause death was not noted. However, another prospective observational study by Ricardo et al. recently reported a thoroughly opposite result that periodontal disease was associated with all-cause mortality, but not cardiovascular death among individuals with CKD [13]. Besides, randomized trials of interventions for periodontal disease in CKD populations are quite scarce and limited by sample size, duration and the use of surrogate indicators [16, 17].

Allowing for its potential but uncertain prognostic effects of periodontal disease in CKD and the need to adopt additional intervention, we therefore conducted a systematic review and meta-analysis to make clear the relevance between periodontal disease and mortality in people with CKD. The effects of diagnostic methods for periodontal disease and the severity of CKD were carefully considered in the present study.

## Methods

This systematic review and meta-analysis is conducted in accordance with the Meta-analysis of Observational Studies in Epidemiology (MOOSE) guidelines [18] and was registered at International Prospective Register of Systematic Reviews (number CRD42016049838).

### Search strategy and selection criteria

Relevant studies were identified by searching the following data sources between 1950 and 1 October 2016: Pubmed, Embase, Scopus, Web of science and abstracts from the 2004–2015 European Renal Association European Dialysis and Transplant Association (ERA-EDTA) Congress. We used Medical Subject Headings (MeSH) and text words of chronic kidney disease, renal replacement therapy, dialysis, kidney transplantation, periodontal diseases and mortality by formulating optimally sensitive search strategies, see Supplementary Search Strategy. Additionally, manual review of reference lists of all included articles was also performed. All potentially eligible studies were retrieved and examined without the limit of languages. If the same cases were reported in more than one study, only the study with the most complete data was included.

### Eligibility criteria

Inclusion criteria: (1) cohort studies that reported the relationship between periodontal diseases and the risk of mortality within adult CKD population, (2) studies in which death risks were evaluated according to periodontal status at baseline, (3) studies in which periodontal status was examined by professional dentists and (4) studies that contained the minimum information necessary to estimate the risk estimates (hazard ratios [HRs], relative risks [RRs]) with 95% confidence interval (CI). Exclusion criteria: (1) cross-sectional studies, (2) studies without estimates of the HRs or RRs of death related to periodontal disease. In addition, no clinical interventions were compared within the included studies.

### Diagnostic criteria of periodontal disease

Measurements for diagnose of periodontal disease mainly included pocket probing depth (PPD), clinical attachments levels (CALs), bleeding on probing, and the alveolar bone status evaluated by radiographic scans. The periodontal clinical examination involving both PPD and CALs measurements was selected as the gold standard, according to the guidelines of the American Academy of Periodontology [19]. Besides, The World Health Organization (WHO) Community Periodontal Index of Treatment Needs (CPITN) is also a validated epidemiological screening tool of periodontal disease [20].

### Diagnostic criteria of CKD

CKD was defined according to the KDOQI guideline [21] (structural or urinary abnormalities with or without glomerular filtration rate < 60 ml/min/1.73m^2^), including people undergoing dialysis and kidney transplantation.

### Data extraction and quality assessment

Two investigators independently screened the titles and abstracts from all eligible studies for relevance (ZJ and JH). Discrepancies between investigators were solved by a third reviewer (SM) until a consensus was reached. In addition, authors were contacted to identify the missing data and make sure issues of interest. Extracted data included the surname of first author, year of publication, study name, study location, stage of kidney disease (non-dialysis-dependent CKD, receiving dialysis, or transplant recipient), study design, sample size, age and gender of participants, duration of follow-up, baseline prevalence of periodontal disease, diagnostic criteria of periodontal disease and CKD, adjusted covariates in multivariable analyses. Adjusted risk estimates (HRs or RRs) were extracted for the following mergence and further analysis. We selected results from the full statistical model that adjusted for the largest number of potential confounders.

The Newcastle-Ottawa scale was used to evaluate the quality of individual studies [22]. In brief, a maximum of 9 points was assigned to each study: 4 for selection, 2 for comparability, and 3 for outcomes. A final score > 6 was regarded as high quality (low risk of bias). Two authors (ZJ and JH) independently extracted the data and gave each study a score. Differences were solved by discussion to achieve consensus, and finally confirmed by another author (SM).

### Outcome measures

We defined the main outcome of interest as all-cause mortality. The secondary outcome was cardiovascular mortality. We extracted risk estimates and 95% CIs (confidence intervals) for outcomes with CKD patients who had no or mild periodontal diseases as the referent category.

### Statistical analysis

All analyses were conducted by using DerSimonian-Laird random-effects and inverse variance model. Multivariable-adjusted risk estimates of dichotomous data were pooled and RRs were presented.

Chi-square test and I^2^ inspection were carried out to determine the heterogeneity between included studies. I^2^ > 50% or Q test *P* ≤ 0.1 were considered to be substantial heterogeneity.

Subgroup analysis was conducted to assess the effect of CKD stage (CKD stage 3–5, CKD 5D, and transplantation), adjustment for confounding variables, study design, study duration, regions, diagnostic criteria of periodontal disease, and reference population.

We performed sensitivity analyses by omitting individual studies stepwise and exploring the effect of individual studies on the overall estimates. Publication bias was evaluated by visual inspection of funnel plot symmetry and Egger’s regression test. All tests were 2-sided and *P* value <0.05 was considered statistically significant. All analyses were performed using STATA version 13.0 software (STATA Corp, College Station, Texas, USA).

## Results

**Fig. 1:**
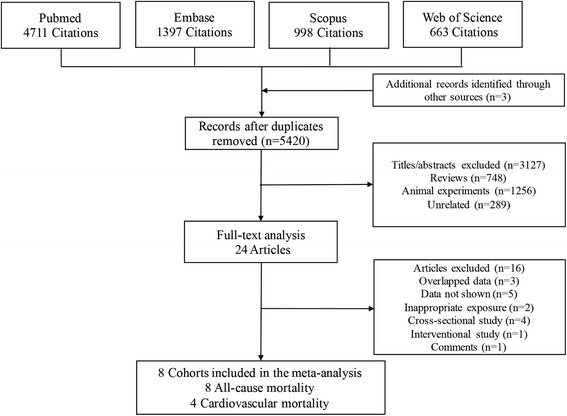
Flow diagram summarizing study identification and selection

We initially retrieved a total of 7772 titles and abstracts. Full-text articles of 24 were obtained for further assessment. We finally identified eight cohort studies in our meta-analysis, including seven prospective cohort studies [13, 15, 23–27], and one retrospective cohort study [14]. Of them, the study by Palmer was reported as a ERA-EDTA conference abstract [23]. Reasons for exclusion of sixteen studies were overlapped data (*n* = 3) [28–30], studies were lack of data (*n* = 5) [31–35], commentary study (*n* = 1) [36], inappropriate study design (cross-sectional study, *n* = 4 [37–40]; intervention study, *n* = 1 [41]), inappropriate exposure (tooth loss) *n* = 2 [42, 43]. Figure 1 illustrates our study selection process.

Four studies used periodontal-disease-free participants as reference group [13, 25–27], three studies enrolled patients with no to mild periodontal disease as reference group [14, 15, 23], and one study used participants with no or moderate periodontal disease as reference group [24]. Only the study by Blach did not adjust for covariant variables [24]. Countries of origin included Europe, United States of America and China. Three studies were populations with CKD stages 3–5 [13, 26, 27], four studies were patients undergoing hemodialysis [14, 15, 23, 25], and one study was of kidney transplant recipients [24]. All of included studies reported diagnostic criteria and methods for periodontal disease. Among them, PPD and CALs were used in five studies [13–15, 25, 26], and CPITN criterion was used in the remained three studies [23, 24, 27]. Data for the risks of mortality were evaluated according to periodontal status at baseline. Characteristics of included studies were summarized in Table 1, with adjusted covariates of each study given in Additional file 1: Table S1. It had been confirmed that no cross-over exists between our included studies.

**Table 1:** Characteristics of included studies

Study	Design	Region	Age (years)	No. of participants (no. of male participants)	Median follow-up duration (years)	CKD stage	Assessment methods and definitions for periodontal disease	Outcome assessment
Blach (26) 2009	Prospective cohort study	Poland	Mean 42.5 years	117 (77)	60 months	Kidney transplantation	CPITN	All cause death
Kshirsagar (15) 2009	Retrospective study	America	Mean 53.6 years	168 (77)	18 months	hemodialysis	PPD, CAL	All cause and cardiovascular death
Chen (16) 2011	Prospective cohort study	Taiwan	58.8 ± 0.8 years	253 (117)	6 years	Hemodialysis	PPD, CAL	All cause and cardiovascular death
Souza (24) 2014	Prospective cohort study	Brazil	Mean 50 years	122 (79)	64.1 ± 11.2 months	Clinically stable patients undergoing HD	PPD, CAL	All cause death
Palmer (28) 2015	Prospective cohort study	Europe and South America	Mean 59.1 years	3338 (1973)	6150 person-years of follow-up	Hemodialysis	CPITN	All cause and cardiovascular death
Ricardo (14) 2015	Prospective cohort study	America	Mean 53.5 years	1335 (544)	14 years	CKD	PPD, CAL	All cause and cardiovascular death
Chen (27) 2015	Longitudinal, observational, community-based cohort study	Taiwan	≥65 years	NA	3.8 years	CKD without ESRD	CPITN	All-cause death
Ruokonen (25) 2016	Prospective cohort study	Finland		144 (97)	157 months	Predialysis	PPD,CAL	All cause death

*Abbreviations: PPD* periodontal probing depth, *CAL* clinical attachment loss, *PDI* periodontal disease index, *CPITN* Community Periodontal Index of Treatment Needs, *BMI* body mass index, *CVD* chronic vascular disease, *CRP* high sensitivity C-reaction protein, *eGFR* estimated glomerular filtration rate, *DMF-T* decayed, missing, and filled teeth index, *NA* not available

Risk of bias in the included studies were summarized in Table 2. We judged 5 [13–15, 25, 26] and 3 [23, 24, 27] of 8 studies to be low or moderate risk of bias overall. The common limitations in the included studies were that age and other important confounders had not been matched between exposure groups and control groups. Besides, follow-up is not long enough for outcomes to occur in some studies.

**Table 2:** Assessment of study quality

Study year	Quality indicators from the Newcastle-Ottawa scale									Score
	Selection				Comparable		Outcome assessment			
	1	2	3	4	5	6	7	8	9	
Blach (26) 2009	*	*	*	*				*	*	6
Kshirsagar (15) 2009	*	*	*	*	*	*	*		*	8
Chen (16) 2011	*	*	*	*		*	*	*	*	8
Souza (24) 2014	*	*	*	*		*	*	*	*	8
Palmer (28) 2015	*	*	*	*			*			5
Ricardo (14) 2015	*	*	*	*			*	*	*	7
Chen (27) 2015		*	*	*		*	*		*	6
Ruokonen (25) 2016	*	*	*	*		*	*	*	*	8

Footnote: For cohort studies, 1 indicates exposed cohort truly representative; 2, non-exposed cohort drawn from the same community; 3, ascertainment of exposure; 4, outcome of interest not present at start; 5, cohorts comparable on basis of age; 6, cohorts comparable on other factor(s); 7, quality of outcome assessment; 8, follow-up long enough for outcomes to occur; and 9, complete accounting for cohorts. Each asterisk represents one star in the Newcastle-Ottawa scale system. The maximum number of stars is 2 for comparability and 1 for each of the other categories, for a total of up to 10 stars

### Periodontal disease and all-cause death in CKD people

**Fig. 2:**
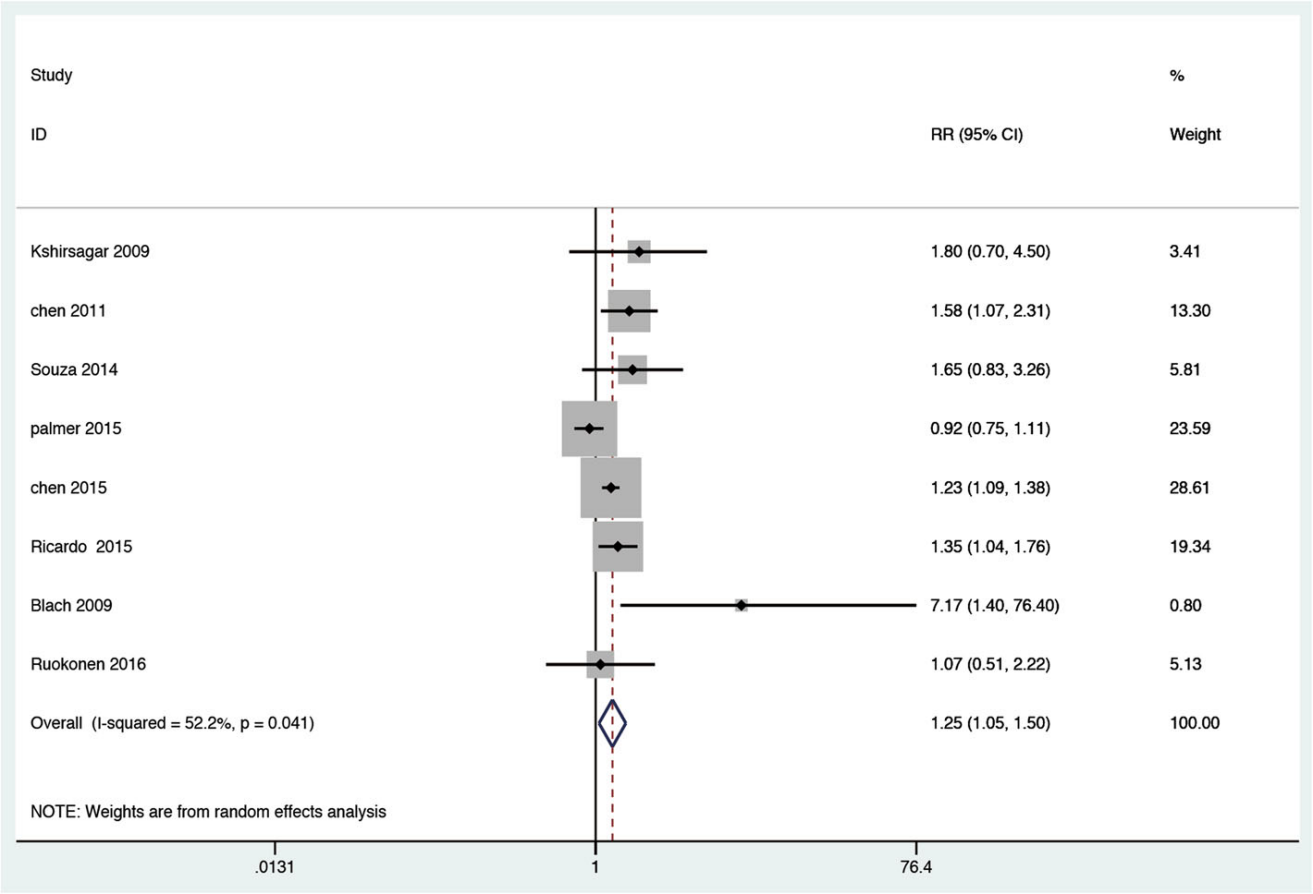
Forest plot of association of periodontal disease and risk of all-cause death in CKD population

A total of eight cohort studies were included in the analysis of all-cause death, comprising 5477 participants and 1492 cases of all-cause death (data from Chen 2015 [27] were not specified). The incidence rate of all-cause death ranged from 3.41% to 46.07%. Overall, there existed a certain correlation between periodontal disease and risk of all-cause mortality in people with CKD (RR, 1.254; 95% CI 1.046–1.503; Fig. 2). Moderate heterogeneity was observed (I^2^ = 52.2%, *P* = 0.041). Therefore, subgroup analyses were conducted to explore sources of heterogeneity. As shown in Table 3, we found that results were similar across different stages of CKD (CKD with renal replacement therapy vs earlier stages of CKD), adjustment for confounding factors, regions, and reference population (healthy population vs healthy to mild periodontal disease population), with *P* values for subgroup difference were 0.79, 0.77, 0.99 and 0.66, respectively. In addition, the extent of adjustment in multivariable analyses did not statistically influence the relationship between periodontal disease and all-cause death as well. However, estimate was slightly modified by the study duration (less than five years vs longer than five years) and diagnostic criteria for periodontal disease. After limiting to individuals with follow-up duration less than five years, increased risk of all-cause death was not observed, with RR = 1.116, 95% CI 0.855–1.457. Due to insufficient data, we were unable to further compare the modality of renal replacement therapy on the association between periodontal disease and all-cause death in CKD population.

**Table 3:** Subgroup analyses for the effects of periodontal disease on all-cause mortality in people with CKD

Overall and subgroup analysis	No. of studies	Heterogeneity		Model	Meta-analysis			*P* value between subgroups
		P	I^2^ (%)		RR	95% CI	P	
Overall	8	0.041	52.2	Random-effect	1.254	1.046–1.503	0.015	
CKD stage								0.79
CKD stage 3–5	3	0.75	0.0%	Fix-effect	1.245	1.119–1.385	0.000	
CKD stage 5D (hemodialysis)	4	0.030	66.4%	Random-effect	1.315	0.885–1.954	0.176	
Transplant	1	NA	NA	NA	7.17	1.4–76.4	NA	
Adjustment for confounding factors								
Unadjusted	1	NA	NA	NA	7.17	1.4–76.4	NA	
Adjusted	7	0.072	48.2%	Fixed-effect	1.194	1.091–1.306	0.016	0.77
Adjusted for smoking and other variables	6	0.058	53.3%	Random-effect	1.209	1.016–1.438	0.032	
Adjusted for diabetes and other variables	6	0.093	47.1%	Fixed-effect	1.175	1.071–1.288	0.001	
Adjusted for diabetes, hypertension and other variables	4	0.664	0.0%	Fixed-effect	1.264	1.137–1.404	0.000	
Study duration								0.14
Short (< 5 years)	3	0.029	71.9%	Random-effect	1.116	0.855–1.457	0.418	
Long (≥ 5 years)	5	0.448	0.0%	Fixed-effect	1.429	1.173–1.742	0.000	
Diagnostic criteria for periodontal disease								0.23
PPD, CALs	5	0.838	0%	Fixed-effect	1.422	1.171–1.727	0.000	
CPITN	3	0.009	78.8%	Random-effect	1.125	0.812–1.559	0.478	
Regions								0.99
Asian countries	2	0.227	31.6%	Fixed-effect	1.257	1.123–1.407	0.000	
European countries and USA	6	0.045	55.8%	Random-effect	1.259	0.927–1.710	0.14	
Reference population								0.66
Healthy	4	0.752	0.0%	Fixed-effect	1.253	1.128–1.393	0.000	
Healthy to mild periodontal disease	4	0.012	72.4%	Random-effect	1.410	0.844–2.356	0.189	

*Abbreviations: RR* relative risk, *CI* confidence interval, *PPD* pocket probing depth, *CALs* clinical attachment levels, *CPITN* Community Periodontal Index of Treatment Needs Index, *NA* data not available

Sensitivity analyses were conducted by excluding individual studies stepwise. Results revealed that relative risks of all-cause death were not remarkably changed, thereby indicating that our results were hardly influenced by small-study effects. The pooled RR was 1.194 (95% CI = 1.091–1.306, *P* = 0.000) when the study did not adjust for any confounders was omitted [24]. While the study by Palmer which was published as a conference abstract was further excluded, the association between periodontal disease and all-cause death in CKD people became stronger, with RR = 1.279, 95% CI, 1.156–1.415, *P* = 0.000. Interestingly, heterogeneity disappeared in the remained studies, with I^2^ = 0.0%, *P* = 0.700. Then we performed a cumulative analysis based on the remained studies in chronological order. Results demonstrated a stable, significant positive association between periodontal disease and all-cause death in people with CKD, shown in Additional file 2: Figure S1. In addition, when we restricted studies to those with low risks of bias, the pooled RR was 1.422, 95% CI, 1.171–1.727, *P* = 0.000, as depicted in Additional file 3: Figure S2.

### Periodontal disease and cardiovascular death in CKD people

**Fig. 3:**
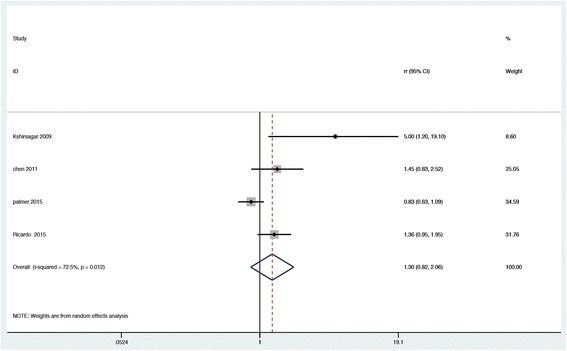
Forest plot of association of periodontal disease and risk of cardiovascular death in CKD population

Of included eight studies, four studies reported the relevance between periodontal disease and cardiovascular mortality [13–15, 23]. Based on a median follow-up of 5.8 years, cardiovascular mortality incidence rates ranged from 8.3% to 23.6%, and the total incidence rate was 13.86%. Random-effects model was utilized to pool corresponding data of individual studies. Results revealed that periodontal disease was not statistically related to the risk of cardiovascular mortality in patients with CKD (RR = 1.30, 95% CI, 0.82–2.06; *P* = 0.259; presented in Fig. 3), with a high heterogeneity (I^2^ = 72.5%; *P* = 0.012).

Subgroup analysis was also performed according to study design, duration of follow-up and reference population. Results indicated that the association between periodontal disease and the risk of cardiovascular death was consistent among subgroups, with *P* values for subgroup difference were 0.23, 0.78, 0.94, respectively.

Eliminating one study at a time, except the study by Palmer [23], all yielded similar effect sizes in magnitude and direction to the overall estimates. But after omitting the study with moderate risk of bias [23], periodontal disease was at a distinctly increased risk for cardiovascular death in CKD people, with pooled RR of 1.469, 95% CI, 1.094–1.972, *P* = 0.011. Moreover, only mild heterogeneity was found in the remained studies, I^2^ = 37.3%, *P* = 0.203.

### Publication bias

**Fig. 4:**
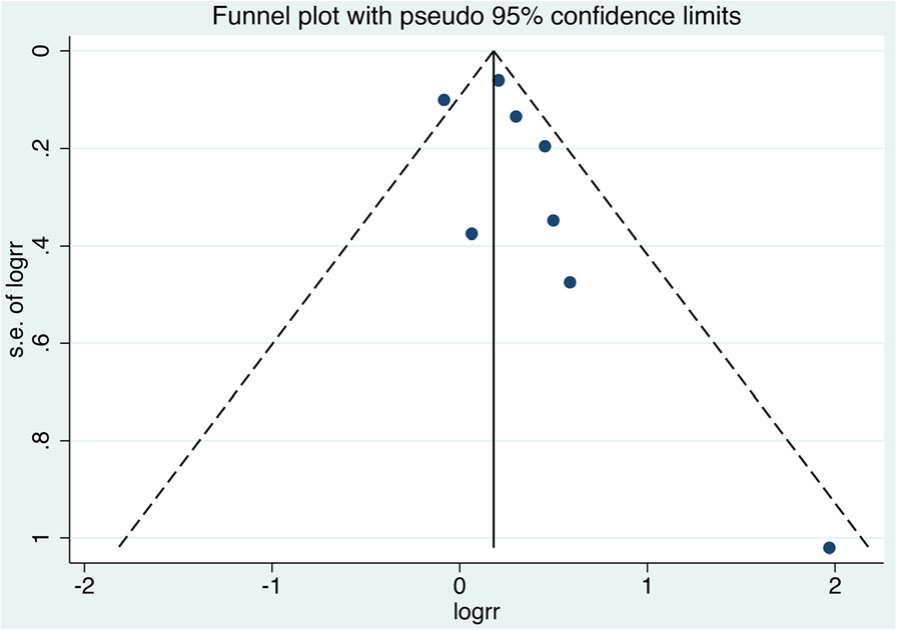
Funnel plot of relative risk of all-cause death in CKD people with periodontal disease

Evidence for publication bias was not noticeable for all-cause death risk on the basis of visual inspection of the funnel plot (Fig. 4) and the Egger’s regression test, with *P* = 0.228. Funnel plot was not conducted for the risk of cardiovascular mortality because of too few included studies, with *P* value for Egger’s regression test was 0.116.

## Discussion

As far as our review team is aware, this study is the first meta-analysis comprehensively evaluating the association of periodontal disease with all-cause and cardiovascular mortality in people who have CKD. A previous systematic review of observational studies by Ruospo et al. have investigated the prevalence and severity of oral disease in adults with CKD, which told us that oral disease is common in CKD people, presented with high incidence of periodontitis, edentulism, mucosal disease, salivary abnormalities and bad oral hygiene [44]. However, the association of periodontal disease with mortality was not meta-analyzed due to the shortage of three independent populations. In our study, the final results manifest that periodontal disease is related with an elevated risk of premature death in CKD population.

Given that smoking is a shared risk factor for periodontal disease and mortality [45, 46], we performed a subgroup analysis by restricting to studies in which smoking status was adjusted, which generated a similar risk of all-cause death. Besides smoking, other confounders such as diabetes mellitus, hypertension and CRP were further analyzed. All kinds of analyses for adjusted estimates suggested a significant relevance between periodontal disease and all-cause mortality. In addition, the association between periodontal disease and death is similar across different races and reference populations. As a whole, these results indicated that periodontal disease could be an independent risk factor of all-cause death in CKD people.

Inconsistency was observed while data were pooled from few studies with follow-up duration less than 5 years. This phenomenon might remind us of the existence of a time lag, with regard to the long-term effect of periodontal disease on the risk of all-cause death in people with CKD.

It is puzzling that meta-analysis of data from large prospective cohort studies showed that CKD people with periodontal disease were not at an increased risk of all-cause death, with RR = 1.146, 95% CI 0.929–1.414. The study by Palmer is one of the three largest cohorts. Adequate evidence revealed that this study, which was based on the ORAL-D cohort study, is the biggest source of heterogeneity among eight included studies. Strangely enough, full-text articles of corresponding data have not yet been published in authority journal, instead, only some related conference abstracts are found [23, 28]. Therefore, it is inferred that the reason for such unstable results pooled from large cohorts lies in the existence of the study by Palmer [23]. Further analysis is limited because we are unable to get more detail information.

Associations between CKD and periodontal disease have been documented in numerous studies [47–49]. Bidirectional relationship between them was confirmed by using a structural modeling methodology, allow for simultaneous modeling of direct and indirect effects [50]. Nevertheless, the exact mechanisms account for the existence of their associations in CKD people are still unknown. Periodontal disease may have a direct causal role in the mortality risks for CKD people. Plenty of evidence manifested that periodontal disease is linked with an increase of several markers of chronic inflammation [33, 51, 52], such as C-reactive protein (CRP), interleukin-6 and fibrinogen. Bacteria involved in periodontal disease can get into the systemic circulation, invade the endothelium of major arteries, then further exacerbate systemic inflammation [53], particularly for hemodialysis individuals, who maintain in chronic persistent inflammation status, per se. As reported that periodontal disease can lead to adverse outcomes in CKD individuals via endothelial dysfunction and vascular injury [54]. A small randomized controlled trial revealed that treatment of periodontitis in dialysis patients improved clinical outcomes of periodontitis severity, but did not generate an obvious impact on serum markers of inflammation [55]. In our present analysis, pooled data from two studies which adjusted CRP levels confirmed the association between periodontal disease and all-cause death in CKD individuals as well.

Another explanation for the increased mortality may be related to poor nutritional status, such as lower albumin and elevated blood urea nitrogen (BUN), Cr levels. Malnutrition is extensively found in CKD population, which impairs the immune defense of the hosts and markedly changes the oral microbial ecology [56, 57]. Through systematic unhealthy nutritional status, CKD can interact with periodontal disease, then contribute to diseases progress together. Previous studies revealed that there existed a significant association between malnutrition and severity of periodontitis [51, 58, 59]. As a consequence, nutrition abnormality can intensify the severity of periodontal disease, which may ultimately develop into life-threatening disease.

Except the potential existence of direct pathways explaining the link between periodontal disease and bad clinical outcomes, it is also plausible that they share similar risk factors in CKD population, such as smoking status, diabetes and hypertension. Corresponding adjusted results were mentioned above, which manifested that other pathogenic mechanisms, other than common risk factor, may take a bigger part in this process.

Different from all-cause death, the definite relevance between periodontal disease and cardiovascular mortality in CKD people is not observed in the present meta-analysis, which excludes the mechanism that elevated all-cause mortality is mediated by cardiovascular disease progression. A large number of publications have suggested that periodontal disease correlated significantly with death from various kinds of cancer [60, 61], diabetes [62], and respiratory disease [63, 64]. In addition, the study by Ruokonen also revealed that noncardiovascular systemic diseases, such as infection, and malignant disease were the main causes of death among CKD populations, which show significant association with periodontal disease [26]. Therefore, these noncardiovascular factors may be suitable to explain the increased risk of all-cause death. Nevertheless, we have to admit that the effects of periodontal disease on the cardiovascular death are less certain, because studies exploring the links between periodontal disease and cardiovascular mortality in CKD are still too few. Larger prospective studies will be useful for better understanding of their association. Hence, we suggest some caution in the interpretation of this result.

### Strengths and limitation

To the best of our knowledge, this meta-analysis is the first of its kind to investigate the controversial issues of whether periodontal disease is associated with all-cause and cardiovascular mortality in CKD population. As some detail data were obtained by contacting with authors, this meta-analysis incorporated some extra unpublished data. In addition, we only included population-based cohort studies, which provide the highest level of evidence in the observational studies.

Limitations of this meta-analysis should be acknowledged, too. Firstly, because of the limited number of original studies and the inevitable methodological limitations of observational studies, our results should be prudently considered as relevant inference and hypothesis-exploring. Just as other results from observational studies, associations do not imply causality certainly.

Secondly, Some studies failed to report the severity of periodontal disease [25, 27], and thus data were unable to allow for the conduct of dose-response analyses.

Thirdly, unadjusted estimate from the study of Blach was also pooled in our meta-analysis.

Fourthly, statistical heterogeneity could not be fully explained after subgroup analyses. We were unable to further investigate the heterogeneity of gender, modalities of renal replacement therapy, method of measuring periodontal disease from other sources, due to the rather small number of included studies.

Fifthly, periodontal disease represents a potentially modifiable risk factor for individuals with CKD. However, longitudinal changes in periodontal conditions during follow up period was not illustrated in almost all studies and it might partially affect the estimates.

Finally, limited information available on periodontal disease and cardiovascular death is also a drawback to our study. Thus this result should still be treated with caution.

## Conclusions

Overall, our findings demonstrated that periodontal disease is significantly associated with an increased risk of all-cause death in people have CKD. However, no adequate evidence proves that periodontal disease is also of an elevated risk for cardiovascular death so far. This meta-analysis of cohort studies incorporated the latest available proof and provided up-to-date insights into the relationship of periodontal disease and mortality risks in CKD people. More high-quality and large scale epidemiological cohorts are required to further establish and verify this association. Additionally, animal studies are also necessary to explore the underlying mechanisms.

## Additional files

Additional file 1: Table S1. Adjustments in studies included in the meta-analysis. (DOCX 75 kb)

Additional file 2: Figure S1. Forest plot of cumulative meta-analysis by adding a single study according to the publication year. (TIFF 250 kb)

Additional file 3: Figure S2. Sensitivity analysis: Forest plot of risk of all-cause death limiting to studies with low risks of bias. (TIFF 252 kb)

## Abbreviations

95% CI: 95% Confidential intervals
BUN: Blood urea nitrogen
CALs: Clinical attachment levels
CKD: Chronic kidney disease
CPITN: Community Periodontal Index of Treatment Needs
CRP: C-reactive protein
HRs: Hazard risks
PPD: Pocket probing depth
RRs: Relative risks
WHO: World health organization
