# Supplementary material for: Association between periodontal disease and mortality in people with CKD: a meta-analysis of cohort studies
Source: BMC Nephrol. 2017 Aug 16;18:269. doi: 10.1186/s12882-017-0680-9 (PMC5558661; doi:10.1186/s12882-017-0680-9)
Supplement: Supplementary file 1 — Adjustments in studies included in the meta-analysis. (DOCX 75 kb) [file 12882_2017_680_MOESM1_ESM.docx]

| **Table S1. Adjustments in studies included in the meta-analysis** | |
| --- | --- |
| **Author (year)** | **Adjustment** |
| Blach (26)  2009 | No |
| Kshirsagar (15) 2009 | Age, center, sex. dialysis vintage, smoking status, cause of end-stage renal disease (ESRD) diabetes mellitus, cause of ESRD hypertension. |
| Chen (16)  2011 | Age; serum albumin level, CRP, Charlson Comorbidity Index score, educational level, and history of smoking. |
| Souza (24)  2014 | Age，sex，diabetes mellitus，hypertension，CRP，frequency of visits to the dentist use of dental floss，DMF-T. |
| Palmer (28)  2015 | Age, sex, race, smoking history, income, medical history (diabetes, myocardial infarction), dialysis vintage, and mean arterial blood pressure, serum phosphorus, and hemoglobin values. |
| Ricardo (14)  2015 | Age, gender, race, education and annual income; diabetes, hypertension, smoking, cardiovascular disease, BMI, family history of premature CVD, HbA1c, total cholesterol and systolic blood pressure. |
| Chen (27)  2015 | Age, sex, BMI, smoking, alcohol use, systolic and diastolic blood pressure, baseline eGFR, hypertension, diabetes mellitus, coronary artery disease, cerebrovascular disease, fasting glucose level, white blood cell count, urinary protein level, and serum total cholesterol, triglyceride, high-density lipoprotein cholesterol, hemoglobin, uric acid, serum urea nitrogen, and albumin levels. |
| Ruokonen  (25) 2016 | Age, smoking status, diabetes, number of teeth, number of medication with mortality. |

Abbreviations: CRP, C-reactive protein; DMF-T, decay missing filled index; BMI, body mass index; CVD, cardiovascular disease; eGFR, estimated glomerular filtration rate.
