# Supplementary figures and images for: Association between periodontal disease and mortality in people with CKD: a meta-analysis of cohort studies
Source: BMC Nephrol. 2017 Aug 16;18:269. doi: 10.1186/s12882-017-0680-9 (PMC5558661; doi:10.1186/s12882-017-0680-9)

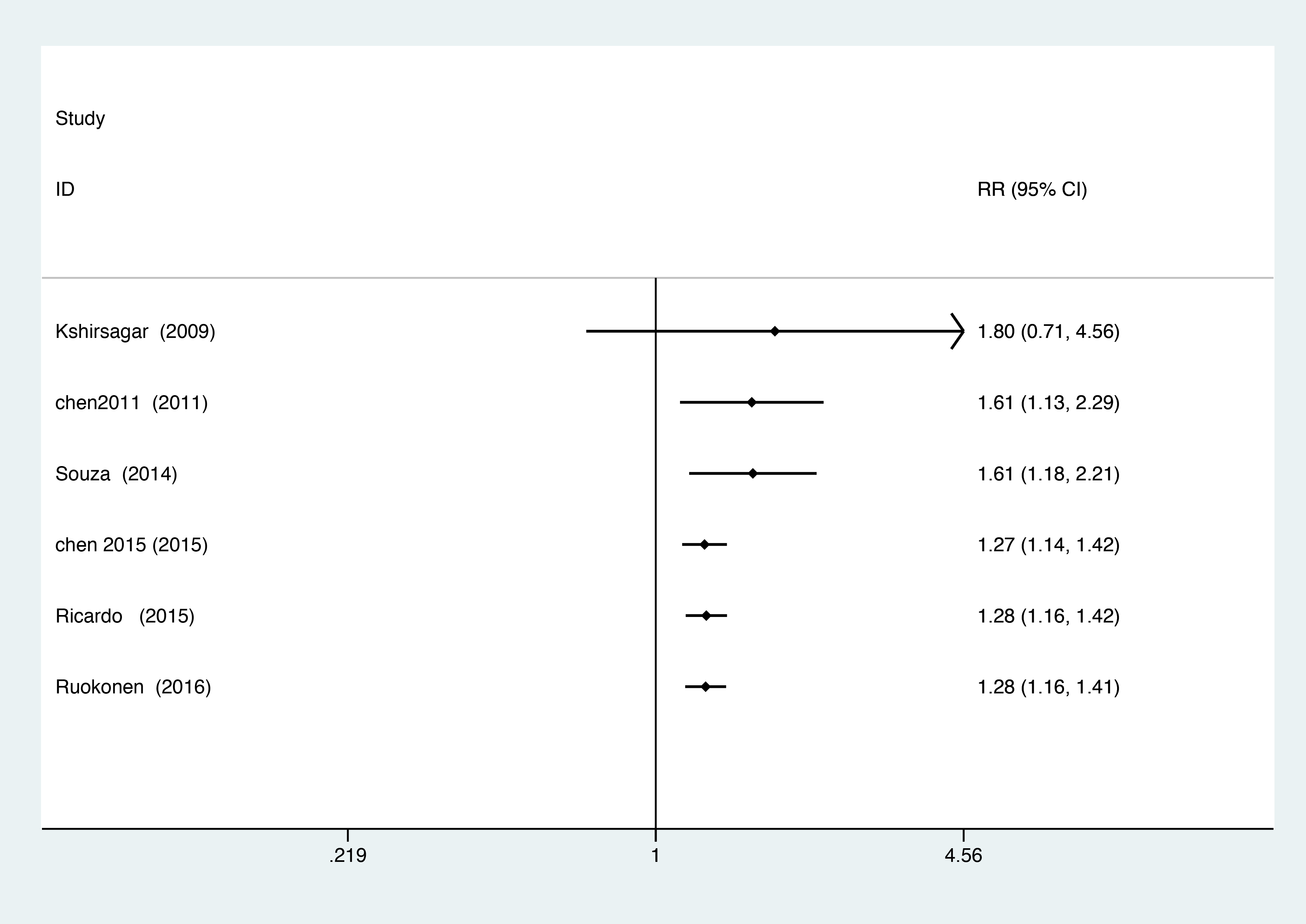

Supplement: Supplementary file 2 — Forest plot of cumulative meta-analysis by adding a single study according to the publication year. (TIFF 250 kb) [file 12882_2017_680_MOESM2_ESM.tif]

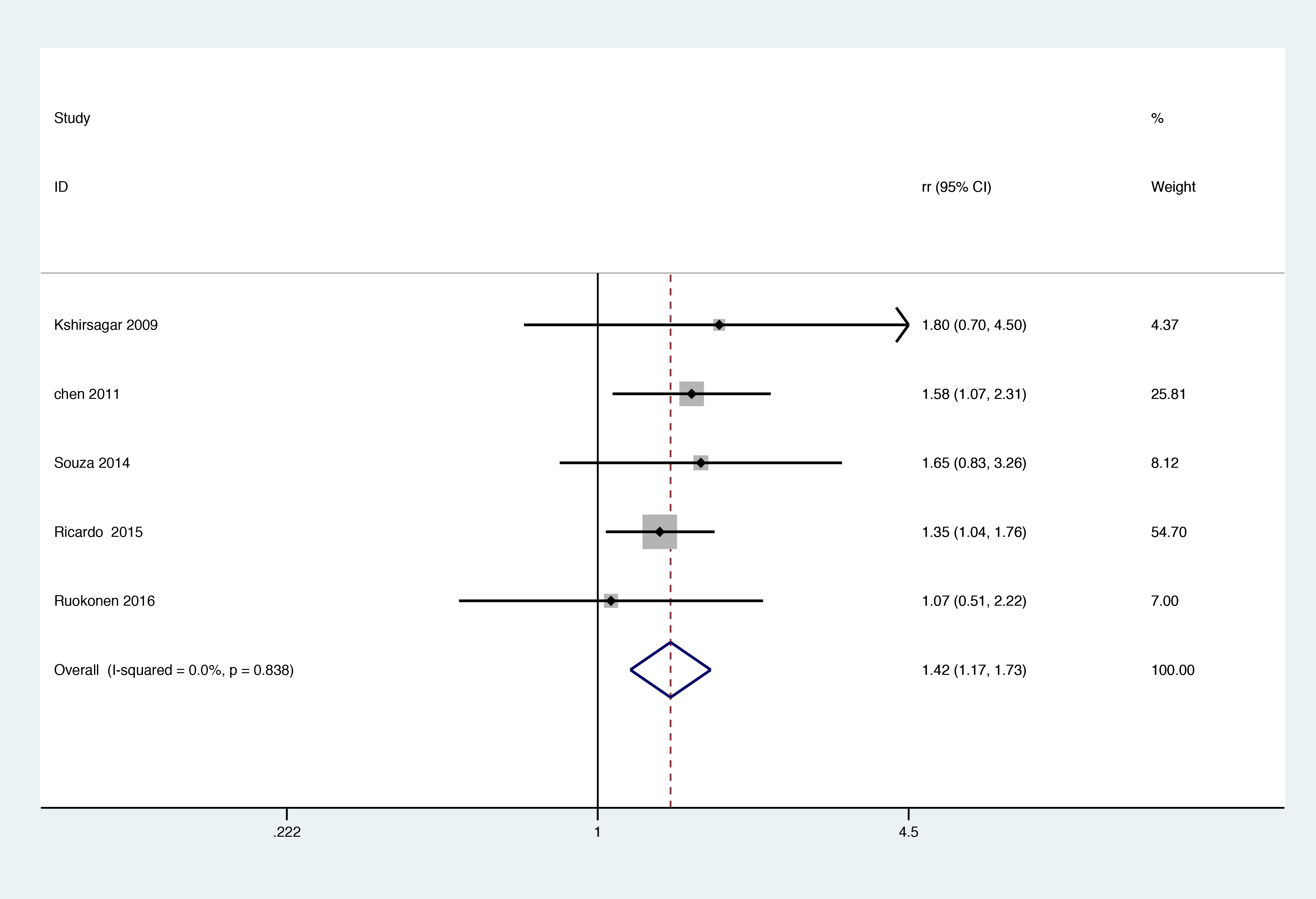

Supplement: Supplementary file 3 — Sensitivity analysis: Forest plot of risk of all-cause death limiting to studies with low risks of bias. (TIFF 252 kb) [file 12882_2017_680_MOESM3_ESM.tif]
